# Mechanical properties of β-HMX

**DOI:** 10.1186/s13065-015-0091-6

**Published:** 2015-04-18

**Authors:** Hugh G Gallagher, John C Miller, David B Sheen, John N Sherwood, Ranko M Vrcelj

**Affiliations:** WESTCHEM, Department of Pure and Applied Chemistry, University of Strathclyde, Thomas Graham Building, 295 Cathedral Street, Glasgow, G1 1XL UK; School of Pharmacy, University of Lincoln, Green Lane, Lincoln, LN6 7DL UK

**Keywords:** Energetics, Hardness, Mechanical properties, HMX, Tensile testing

## Abstract

**Background:**

For a full understanding of the mechanical properties of a material, it is essential to understand the defect structures and associated properties and microhardness indentation is a technique that can aid this understanding.

**Results:**

The Vickers hardness on (010), {011} and {110} faces lay in the range of 304–363 MPa. The Knoop Hardnesses on the same faces lay in the range 314–482 MPa. From etching of three indented surfaces, the preferred slip planes have been identified as (001) and (101). For a dislocation glide, the most likely configuration for dislocation movement on the (001) planes is (001) [100] (|b| = 0.65 nm) and for the (101) plane as (101) $\left[10\overline{1}\right]$ (|b| = 1.084 nm) although (101) [010] (|b| = 1.105 nm) is possible. Tensile testing showed that at a stress value of 2.3 MPa primary twinning occurred and grew with increasing stress. When the stress was relaxed, the twins decreased in size, but did not disappear. The twinning shear strain was calculated to be 0.353 for the (101) twin plane.

**Conclusions:**

HMX is considered to be brittle, compared to other secondary explosives. Comparing HMX with a range of organic solids, the values for hardness numbers are similar to those of other brittle systems. Under the conditions developed beneath a pyramidal indenter, dislocation slip plays a major part in accommodating the local deformation stresses.

**Graphical abstract:**
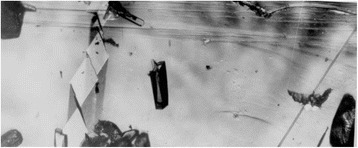
HMX undergoing tensile testing.

**Electronic supplementary material:**

The online version of this article (doi:10.1186/s13065-015-0091-6) contains supplementary material, which is available to authorized users.

## Introduction

To develop a firm understanding of the mechanical properties of any given material, it has been established that in addition to understanding the crystal structure, a knowledge of the defect structures and their properties is also essential [1,2]. To study the defect structure of a given material, methods such as etching and x-ray topography can be used to identify dislocations, their density and type. However, to fully understand the interrelationships between the defects and the material that they inhabit, more direct methods of mechanical deformation must be used. Microhardness indentation is a useful technique for such an investigation, as it can yield a great deal of information from a careful study. In recent years more interest has been shown in the application of this method in the field of organic materials, especially pharmaceuticals [3-5] and explosives [6,7]. In these cases the understanding of the mechanical properties can be used to inform and influence the control of such processes as milling, granulation and comminution. All these common processes involve mechanical deformation of organic materials; however the relationships between the mechanical deformation and processing demands are in general poorly understood. A missing key to improving this understanding is the better definition of the defect structure of organic solids and how this reacts to a mechanical stimulus. Mechanical deformation by indentation can be extremely useful in defining the mechanical properties that any given material may exhibit with a particular focus on the material surface. In addition to point loading studies, tensile and compressive deformation [8] studies of whole crystals can give a more general understanding of the processes occurring within the bulk of a crystal.

In the particular case of energetic materials, such studies are important as they can contribute to a more complete understanding of self-detonation and stability. It is now established [9-11] that dislocations can move and “pile-up” against either an obstacle or each other. This energy localization due to plastic deformation (hot-spots) can significantly affect the detonation initiation process. While hot spots created by purely defect based methods are sub-critical in nature, they will add to those that can be critical. Such studies have already been performed on energetic materials such as Cyclotrimethylene Trinitramine (RDX) [6,7,12-14], Pentaerythritol Tetranitrate (PETN) [6,7,12,15,16], where deformation by slip is important and by a topographic defect study of 2-4-6 Trinitrotoluene (TNT) [17] where growth twinning and polymorphism influences the defect structure. Less attention has been paid however to the commonly used explosive Cyclotetramethylene-Tetranitramine (HMX).

The most stable form (at room temperature) of HMX (C_4_H_8_N_8_O_8_) is the β-form [18-21], this is monoclinic, space group P2_1_/n, a_0_ = 0.6525 nm, b_0_ = 1.1037 nm, c_0_ = 0.7364 nm, β = 102.66° with one molecule in the asymmetric unit. The packing is dominated by Van der Waals forces, as there are no appropriate donor/acceptor groups to establish any hydrogen bonding network. A cleavage plane exists parallel to (011) [22].

Although β-HMX has been studied thoroughly as to its explosive properties, only a small amount of work has been performed in relation to its defect properties and mechanical properties. Work by Amuzu et al. [23] reported a study on the frictional properties of a set of energetic materials and obtained a Vickers Hardness Number (VHN) of 405 MPa for β-HMX although they did not specify the indented face or crystal orientation. Palmer and Field [22] studied the deformation and fracture behaviour of β-HMX by compression, wettability and indentation and etching (Vickers indentations on a (010) face). They showed that indentation resulted in the formation of both mechanically induced twin lamellae on (101) planes and cracks on {011} cleavage planes. By measuring the size of the surface crack as a function of indenter load, the authors determined the fracture surface energy. They also reported the VHN for indentation as 395 MPa and etched the indented face to reveal dislocation activity around the Vickers impressions. A later investigation by Palmer et al. [24] into the general mechanical properties of β-HMX embedded as a plastic bonded explosive has also been reported, and a detailed examination in part of the present study [25] has shown by etching methods that growth dislocations emergent on the (010) face are of either edge (∣b∣ = [010]) or screw (∣b∣ = [010]) type. Better understanding of the contribution of phonons and lattice vibrations to detonation via elastic properties have been made experimentally [26,27] and with supporting computational studies [28,29]. In addition a nanoindentation study [30] has indicated that the hardness of the (010) face of β-HMX is ~1080 MPa, which is much higher than that found in previous studies. While the nanoindentation studies are extremely useful and suggests the mechanisms of deformation as slip, these studies do not full characterize the crystal, with only the (010) face identified and measurements being reported on a second unidentified face. However, these do give an estimate of the elastic modulus of the known face.

In this work, previous studies of the mechanical deformation of this material are being extended to include all three predominant habit faces of β-HMX. Both Vickers and Knoop indenters have been used to investigate variations of hardness with orientation, load and temperature. Indentation followed by etching has been employed to identify the active slip systems, thereby allowing comparison of the measured hardness with the hardness variation with crystallographic orientation predicted by effective shear stress calculations. In addition, preliminary examinations of tensile testing has been used to demonstrate the mechanical behaviour of β-HMX under cyclical processing.

### Experimental

The HMX was supplied by PERME, Waltham Abbey. Crystals of β-HMX were grown by evaporation at room temperature from unseeded acetone (purity 99%+, obtained from Sigma-Aldrich) solutions [25].

Microhardness Indentation: Measurements were carried out on the as grown (010), {011} and {110} faces using a Leitz Miniload microhardness tester, fitted with either Vickers or Knoop pyramidal indentation heads. For all faces Vickers indentation measurements were made as a function of crystal orientation and load. In addition, the hardness of the (010) face was measured as a function of temperature using an in-house designed heating stage. Knoop indentation measurements were made as a function of crystal orientation and an average value of at least 10 measurements were taken at each orientation. Post indentation etching was carried out using pure acetone at 293 K and an etch time of 1 second [25].

Tensile tests: These were carried out on HMX using a tensile stage as described by Bowen and Miltat [31]. The stage measures about 50 mm × 100 mm and flat specimens of size 10 mm × 5 mm can be mounted in the sliding carriage. The carriage runs on precision linear roller bearings in order to maintain the specimen mounting plates coplanar and thus minimise specimen bending during tension. The stage is capable of applying a stress of 20–5000 MPa. The load is applied to the specimen via a spring-steel cantilever which bears on the sliding carriage. The tensile force is measured by a strain gauge bonded to the cantilever and the output from this is fed to a control box which gives a direct digital read-out of tensile force in Newtons. The cantilever spring is operated by a micrometer screw driven by a small 12 V D.C. motor.

## Results

### Indentation of (010) faces

**Figure 1:**
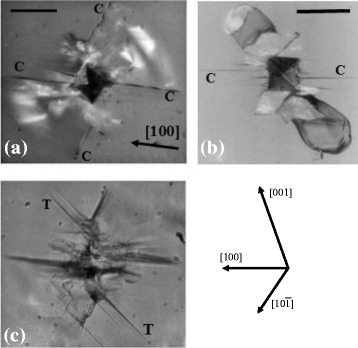
Reflection optical micrographs of Vickers indentations. On the (010) surface of HMX with the horizontal indenter diagonal **(a)** parallel to and **(b)** at 45° to [100]. **(c)** Indentation **(a)** in transmission. (T) twin lamellae, along $\left[10\overline{1}\right]$ and (C) cleavage cracks. Scale bar 40 μm.

The Vickers hardness was determined as a function of indenter orientation, load and temperature. In the first orientation, one of the indenter diagonals was aligned parallel to [100] whereas in the second it was positioned at 45° to this direction. The resulting VHNs when using a load of 15 g are presented in Table 1 and examples of the resulting hardness impressions in Figure 1. At each orientation, the surface traces of cracks lying parallel to [100] are observed. These correspond to cleavage cracks on {011} planes. At the first orientation only (Figure 1a), irregular cracks extend from one of the indenter diagonals for a short distance before diverging to lie along the $\left[10\overline{1}\right]$ direction. In Figure 1(a) the initial part of the crack, before divergence, is hidden by the optical distortion close to the indentation. In the second orientation similar short $\left[10\overline{1}\right]$ direction cracks initiate from the tips of the indentation {001} diagonal and cracking also occurs in a plane parallel to the surface due to partial cleavage on the (010) planes. Examination of the indentations using transmission optical microscopy (Figure 1c) reveals a number of twin lamellae lying parallel to one another and normal to the $\left[10\overline{1}\right]$ direction. Far more twins are produced for the second orientation of the indenter than for the first.

**Table 1:** **Variation of VHN at different orientations on the (010), {011} and {110} faces of β-HMX**

**Face**	**Direction**	**Indenter orientation from direction (°)**	**VHN (MPa)**
(010)	[100]	0	362.8
(010)	[100]	45	323.6
{011}	[011]	0	313.8
{011}	[011]	45	343.2
{110}	[001]	0	304.0
{110}	[001]	45	313.8

**Figure 2:**
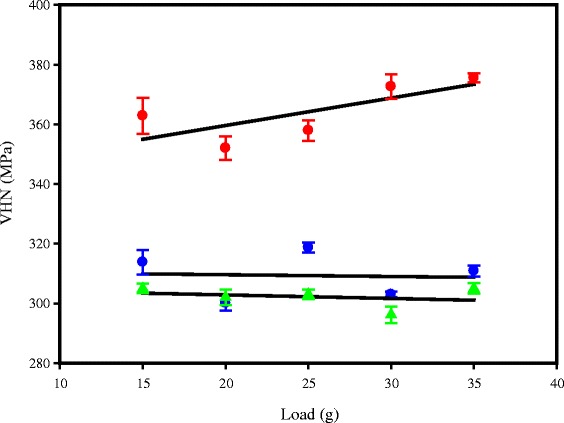
The variation of VHN with load: Red circles indicate the (010) face; blue circles indicate the {011} faces and green triangles the {110} faces.

The load dependence of Vickers hardness was determined using loads in the range 15-35 g. The orientation of the indenter was maintained constant for all of the measurements corresponding to a position with one diagonal parallel to the [100] direction. The subsequent plot of VHN against load (Figure 2) shows that the hardness is essentially independent of load. At higher loads, size of both cracks and twin lamellae increased, with no change in orientation. However at loads greater than 35 g, the deterioration in the hardness impression due to severe cracking made accurate measurements of VHN impossible.

**Figure 3:**
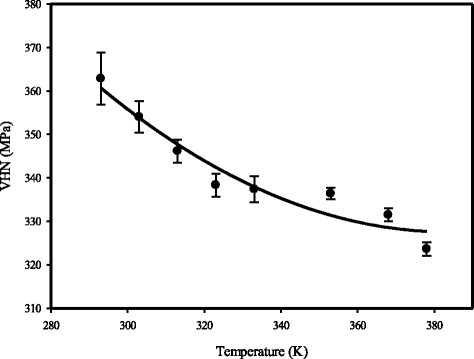
The variation of VHN with temperature on the (010) face. The line for guidance only.

The effect of temperature on hardness was assessed by indentation at various temperatures in the range 293-378 K using a specially designed heating stage mounted on the hardness tester. A load of 15 g was employed and an orientation as above with a diagonal parallel to the [100] direction. The results presented in Figure 3 show the hardness to decrease gradually with increasing temperature up to 378 K, which is to be expected for a material which is dominated by Van der Waals bonding.

**Figure 4:**
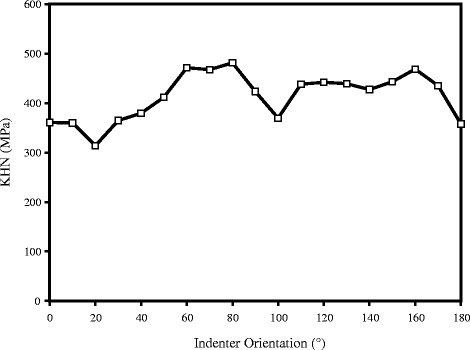
Variation of KHN with orientation of the long diagonal on the (010) face of β-HMX [100] = 0°.

**Figure 5:**
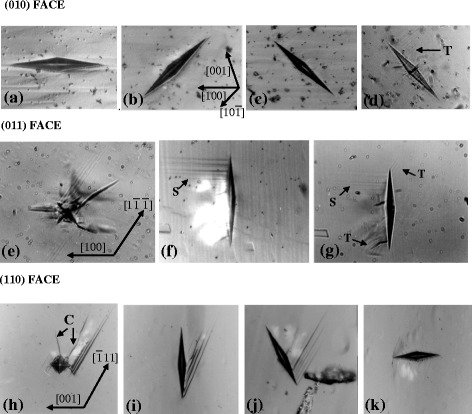
Indentations on the β-HMX crystal habit faces. {010} Face: **(a)** Knoop, long diagonal parallel to [100] (Ref.); **(b)** Knoop, long diagonal parallel to $\left[10\overline{1}\right]$ (Ref.); **(c)** Knoop, long diagonal normal to $\left[10\overline{1}\right]$ (Ref.); **(d)** Knoop, long diagonal normal to $\left[10\overline{1}\right]$ (Trans). {011} Face; **(e)** Vickers, diagonal 45° to [100] (Trans); **(f)** Knoop, normal to [100] (Ref); **(g)** Knoop, normal to [100] ((f) in Trans). {110} Face: **(h)** Vickers, diagonal parallel to [001] (Ref); **(i)** Knoop normal to [001] (Ref); **(j)** Knoop, long diagonal 65° to [001] (Ref); **(k)** Knoop parallel to [001] (Ref). S = Slip traces, T = twin bands, C = cracking, (Ref.) and (Trans.) = reflection or transmission photomicrograph.

The Knoop hardness number (KHN) was determined as a function of indenter orientation only. Indentations were made with the long diagonal aligned at successive 10° intervals in the range 0-180° with respect to the [100] direction using a 15 g load. The results are summarized in Figure 4. In contrast to the Vickers indentations, the Knoop indentations are more precise and the extent of both brittle fracture and twinning is reduced. This is illustrated in a series of optical micrographs of differently oriented Knoop indentation impressions (Figure 5(a) – (d)). As already shown with the Vickers indentations, the extent of twinning varies significantly depending on the indenter orientation. Indentations made with the long diagonal of the indenter normal to the $\left[10\overline{1}\right]$ direction exhibit a relatively high density of twin lamellae, whereas fewer twins are formed at indentations aligned along [100] and none at all are observed at indentations oriented parallel to the $\left[10\overline{1}\right]$ direction (Figure 5(b)).

### Indentation of the {011} face

On the {011} habit face, Vickers indentations were made at two different orientations, one with a diagonal parallel to [100] and one with a diagonal at 45° to this direction, using a load of 15 g. Hardness measurements are presented in Table 1 and a typical hardness impression is shown in Figure 5(e). Once again extensive cracking is associated with the orientations. The majority of cracks appear randomly lying along high index crystallographic directions. The exceptions are those that occur on the {011} cleavage planes, whose surface trace extends in the [010] direction parallel to the (100) planes. The transmission optical micrographs again reveal a series of mechanically induced twin lamellae lying on the (101) planes. The extent of twinning is greater at indentations with the diagonal oriented at 45° to [100] (shown in Figure 5(e) than for those oriented parallel to this direction. The load dependence of the Vickers hardness number was once again determined using loads in the range 15-35 g with the indenter oriented with a diagonal along [100]. These results are presented in Figure 2. As with the (010) face, the VHN is found to be independent of load in the range employed and indentations at higher loads resulted in a corresponding increase in both crack length and twinning.

The Knoop hardness number was measured as a function of orientation by indenting parallel to a number of important crystallographic directions using the 15 g load. These Knoop hardness results are given in Table 2. As with the (010) face, less cracking and twinning occurred around the Knoop indentations compared with the Vickers and the extent of twinning was strongly dependent on the orientation of the indenter. Photomicrographs of a typical indentation are given in Figures 5(f) and (g). In addition to the occurrence of twins there are, what appears to be, slip traces running parallel to [100]. These are characteristic of localized plastic deformation by dislocation slip. They occur around indentations oriented perpendicular to the [100] direction and lie parallel to the surface intersection of the (101) planes.

**Table 2:** **KHN on {011} and {110} faces of β-HMX as a function of orientation**

**{011}**	
Indenter orientation from [100] (°)	KHN (MPa)
0	372.7
31	343.2
90	343.2
122	353.0
**{110}**	
Indenter orientation from [001] (°)	KHN (MPa)
0	451.1
57	372.7
90	382.5

### Indentation of {110} face

The Vickers indentations were performed with an indenter diagonal oriented parallel and at 45° to the [001] direction and a load of 15 g are also shown in Table 1. The hardness impression shown in Figure 5(h) exhibits both extensive fracture and twinning. Since the twins emerge onto the surface producing a step, they can be viewed in reflected light. The extent of twinning varied only slightly with indenter orientation. The cracking is irregular, does not follow preferred crystallographic directions and is distributed almost radially around the indentations.

The variation of VHN with load was again determined only in the limited range of 15-35 g with an indenter diagonal parallel to [001] (Figure 2). Within this range, the Vickers hardness is effectively constant. At increased loads the deformation patterns become proportionally larger, but maintain the same overall appearance.

The KHN was determined at various significant crystallographic orientations using a 15 g load. The hardness results are again presented in Table 2 and a number of Knoop impressions are shown in Figure 5(i) – (k). Although there is less cracking in comparison with Vickers indentations, the degree of twinning is still very high and is orientation dependent.

### Dislocation etching around indentations

Following indentation on the three major habit faces of β-HMX, the samples were etched in order to examine the extent of plastic deformation associated with the indentations. The active slip systems were determined and the densities and configurations of the dislocations within the slip plane assessed. In order to resolve individual etch pits associated with the indentations, it was necessary to reduce the etching time previously defined to approximately one second.

**Figure 6:**
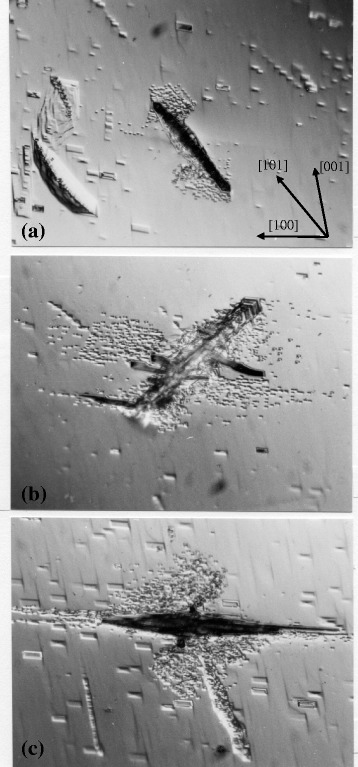
Etched Knoop indentations on a (010) face. **(a)** Long diagonal parallel to [101], load 15 g; **(b)** Long diagonal perpendicular to $\left[10\overline{1}\right]$, load 25 g; **(c)** Long diagonal parallel to [100], load 25 g.

Figure 6 shows a number of etched Knoop indentations at various orientations on the (010) face. Rows of dislocation etch pits extending along well defined crystallographic directions are observed around the indentation impressions. Two distinct etch pit alignments are visible, corresponding to the intersection of dislocation slip planes with the (010) surface. These lie parallel to the [100] and [101] directions and their presence depends critically on the orientation of the indenter. Close examination of the etch pit morphology under high magnification revealed the pits to be identical to the type 1 pits described in an earlier communication [25] and are thus the ends of edge dislocations with b = [010]. The apex of each pit is coincident with the geometric centre, indicating that the dislocation lines are normal to the crystal surface. This defines the slip planes as (001) and (101). Therefore, the greater density of etch pits at the sites of indentation arises from stress induced dislocation loops which are contained in the (001) and (101) slip planes and emerge normal to the (010) face.

**Figure 7:**
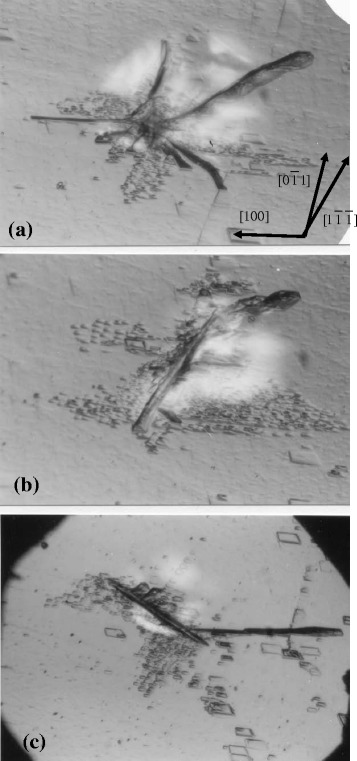
Etched indentations on a {011} face.** (a)** Vickers indentations with diagonal parallel to [100]; **(b)** Knoop indentation with long diagonal parallel to the surface intersection of the (101) plane, load 25 g; **(c)** Knoop indentation with long diagonal perpendicular to the surface intersection with the (101) plane, load 25 g.

The distribution of etch pits found around Vickers and Knoop indentations on {011} faces is shown in Figure 7. Two alignments are clearly seen, corresponding to the surface traces of (001) and (101) planes, confirming the observations made on the (010) habit face. A more detailed inspection of the etch pit morphology showed that the pits that appear along the two slip traces are identically shaped, with their apex similarly displaced from the pit centre. This type of trapezoidal pit has also been described in a previous communication [25], although the Burgers vector is as yet unidentified. The only crystallographic direction common to both slip planes is defined by their line intersection as [010]. In addition, all the pits exhibit the same oblique orientation with respect to the surface irrespective of their position around the indentation mark. This suggests that the etch pit alignments are derived from dislocation loops lying in the (001) and (101) slip planes whose emergent ends are parallel to the [010] direction.

**Figure 8:**
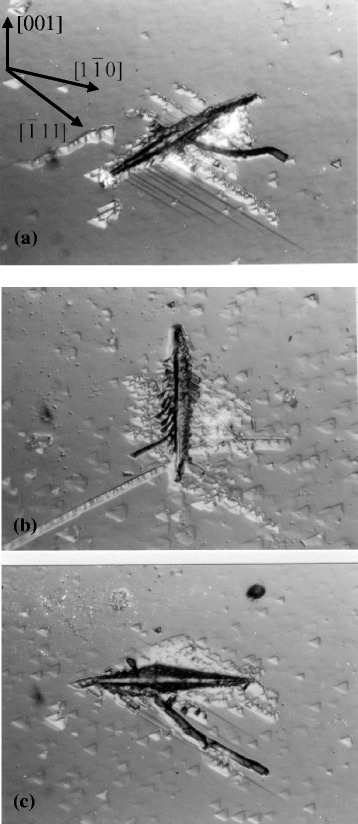
Etched indentations on a {110} face.** (a)** Long diagonal almost normal to the surface intersection with the (101) plane, load 25 g; **(b)** Long diagonal parallel to [001], load 25 g; **(c)** Long diagonal normal to [001], load 25 g.

Etched Knoop indentations on the {110} surface obtained using the 25 g load are shown in Figure 8. They show only one etch pit alignment, irrespective of indenter orientation, which is parallel to the intersection of the (101) plane with the {110} surface. No alignments corresponding to the surface trace of the (001) plane are apparent. All of the pits are highly asymmetric pentagons (again as described previously [25]) suggesting that they reveal the ends of inclined dislocations. As the pits in a single alignment on both sides of the indentation show equivalent asymmetry, they must represent the surface intersection of asymmetrically shaped dislocation loops. The etch pits are quite distinct from the twin lamellae and so the dislocations revealed clearly cannot be involved in the twinning process.

From the geometry and alignment of the pits on the three indented and etched surfaces, the preferred slip planes have been identified as (001) and (101). For a dislocation to move by glide, the Burgers vector must be parallel to the slip direction. A list of lattice planes and interplanar spacings is given in Table 3. Since the energy of a dislocation is proportional to |b|^2^, the most likely configuration for dislocation movement on the (001) planes is (001) [100] (|b| = 0.65 nm). Similar arguments lead to the most likely configuration on the (101) plane as (101) $\left[10\overline{1}\right]$ (|b| = 1.084 nm) although (101) [010] (|b| = 1.105) appears a distinct possibility.

**Table 3:** **Interplanar spacing for low index planes and Burgers vectors |** **b** **| in β-HMX**

**Planes h, k, l**	**Interplanar spacing d (nm)**
011	0.602
110	0.552
020	0.552
$10\overline{1}$	0.539
$1\overline{1}\overline{1}$	0.484
021	0.438
101	0.432
002	0.359
b	|b| (nm)
[100]	0.654
[001]	0.736
[101]	0.873
$\left[10\overline{1}\right]$	1.084
[010]	1.105

As the emergent dislocations in both slip systems on a (010) face are normal to the crystal surface, and both the [100] and $\left[10\overline{1}\right]$ Burgers vector lie in this plane, etch pits associated with this face correspond to the intersection of pure edge components of the dislocation loops. On a {011} face, the emergent slip dislocations are parallel to [001]. As both [100] and $\left[10\overline{1}\right]$ directions are perpendicular to this direction, the etch pits observed are also associated with pure edge segments of dislocation loops which lie in either the (001) or (101) planes, although in the case of the latter, the Burgers vector is not parallel to the (001) surface. It is not possible to speculate about the character of the emergent slip systems from information from indentations on a {110} face since the dislocation line direction cannot be determined from the etch pit geometry.

### Effective resolved shear stress calculations

The nature of the deformation process associated with Knoop indentations of β-HMX is complex and involves slip, twinning and to a minor extent brittle fracture, often occurring simultaneously. Despite this, an attempt has been made to explain the hardness anisotropy and give support to the conclusions drawn from etching studies regarding the operative slip systems by using the simple theoretical model developed by Daniels and Dunn [32] and the later modifications of Brookes, O’Neill and Redfern [33] (information about the method of calculation are given in the Additional file 1 to this paper). The potential for attempting such calculations in the present circumstances is well defined by the success of previous similar examinations on the energetic materials PETN and RDX [7] and the inorganic materials calcite and sodium nitrate [34]. This model is based on explaining the orientational dependence of Knoop hardness on the effective shear stress (ERSS) acting on each slip system during indentation. The KHN is related to the extent of slip predicted by the ERSS calculations, assuming only one slip system to be active at any one particular orientation of the indenter. In a situation where more than one slip system with comparable activities is available, it has been shown that only the slip system with the greatest ERSS value is activated. Also that as a consequence, a change from one slip system to another can occur during successive orientations of the indenter when the ERSS value of the second system exceeds that of the first. Although the treatment has been applied successfully to explain anisotropy in Knoop hardness for many materials on the basis of operative slip systems, its application to twinning has rarely been considered. However calculated ERSS curves for known twinning systems show a good correlation with the Knoop hardness anisotropy for single crystals of calcite [34] and magnesium [35] where the deformation is dominated by twinning. It is not possible to judge the relative importance of slip and twinning in the deformation of HMX, under the conditions encountered during indentation from purely microscopic observations around the impressions. Twinning must therefore be considered when comparing the hardness anisotropy and the ERSS results as it is a potentially important deformation mechanism. A major problem arises since the potential (101) $\left[10\overline{1}\right]$ slip system involves the same plane and direction as that proposed for twinning, and so difficulty differentiating between the two might be expected.

As mentioned earlier, HMX has no strong KHN anisotropy with change of crystal orientation on any of the surfaces studied, so in this case a full analysis was only carried out on the (010) face, where the slip systems are assumed to be (101) $\left[10\overline{1}\right]$ and (001) [100]. In this particular case, the Brookes ERSS model has to be used, as the configuration would lead to a Daniels and Dunn prediction of absolutely no variation of ERSS with orientation.

**Figure 9:**
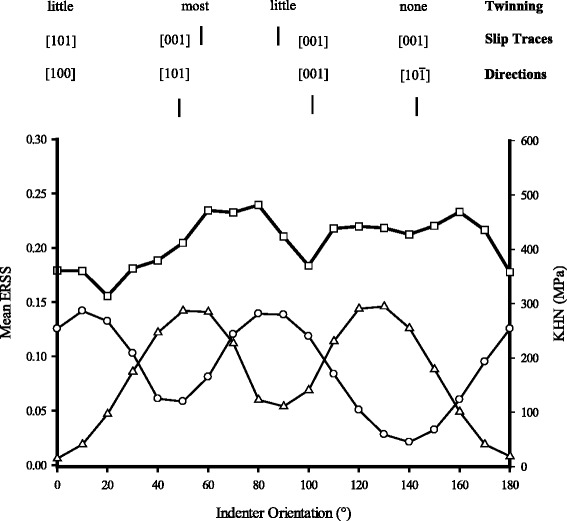
Comparison of the experimental hardness measurements (□) with ERSS calculations for (101) $\left[10\overline{1}\right]$ (○) and (001) [100] (△) slip systems: Observational data on slip and twinning is given for specific orientations [100] = 0°.

In Figure 9, the experimental hardness measurements taken on the (010) face are compared with the ERSS values for the (101) $\left[10\overline{1}\right]$ and (001) [100] systems. Included on this graph are the microscopic findings specifying the active slip system and extent of twinning at specific orientations. For the {110} and {011} faces, the results are presented and compared with the hardness measurements and photomicrographic information in Table 4.

**Table 4:** **Knoop indentations on {011} and {110} faces: Comparison of observed slip traces and extent of twinning with the ERSS calculations for (001) [100] and (101)** $\left[10\overline{1}\right]$ **systems**

		**ERSS**			
**Crystal Face**	**Orientation (°)**	**(001) [100] A**	**(101)** $\left[10\overline{1}\right]$ **B**	**Observed slip traces**	**Twinning**
{011}	0 [100]	0.0043	0.0775	B	Very little
	31	0.0533	0.0956	B	Very little
	90	0.3261	0.2336	A	Some
	122	0.1767	0.1140	A	Some
{110}	0 [001]	0.0677	0.0533	B	Very little
	65	0.2443	0.1890	B	Rel. extensive
	90	0.3091	0.2215	B	Rel. extensive

The lack of relatively large hardness anisotropy for the (010) face is reflected in the ERSS curves if it assumed, as the theory demands, that only the system experiencing the maximum ERSS is activated at a given orientation. The observations of dislocation etching point to which systems are operative at any given orientation and clearly confirm this premise. A maximum in the ERSS requires a minimum in the hardness and so bearing in mind the potential complexity of the processes involved, there would appear to be an acceptable correlation between the experimental curve and the ERSS values. It is unlikely that much can be deduced from the positions of minor minima and maxima in these plots.

Twinning, although of apparently secondary importance, presents something of an anomaly. The results of Palmer and Field [22] suggest that the process is activated by a shear on the (101) plane along the [101] direction and this has been confirmed by our own results on the tensile deformation of large single crystals [36]. However, little twinning is observed at orientations where calculations predict a maximum value of the effective resolved shear stress, suggesting that under the conditions experienced during indentation on this face, twinning is not brought about by the tensile component of the shearing force in the [101] direction.

As for indentation on the {011} face, the experimental hardness shows very little variation with orientation in contrast to the ERSS values calculated for the (101) $\left[10\overline{1}\right]$ and (001) [100] slip systems. However the small changes that are observed do show a similar trend to the ERSS values and the observed slip traces are those predicted on the basis of theory. Twins are observed at all orientations studied, but do show a greater presence when the ERSS for the (101) $\left[10\overline{1}\right]$ system is also high.

On the {110} face, the hardness results again show little variation with orientation, although there are significant changes in the ERSS values. The trend is again for a higher KHN where the ERSS values are low. However, only one type of slip trace is observed over the whole range; that associated with the direction of interception of the (101) plane. This is in spite of the fact that ERSS values calculated for the (001) [100] system are significantly higher than those for the (101) $\left[10\overline{1}\right]$ system. Also twinning is observed to be very extensive and dependent on orientation.

### Tensile testing

**Figure 10:**
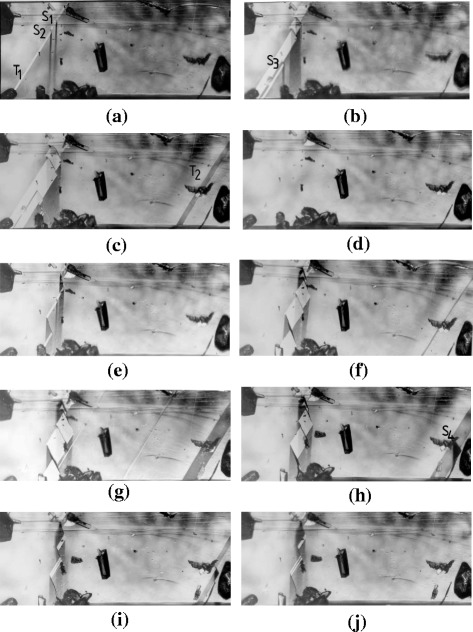
Tensile testing images.

The twinning behaviour at various values of tensile stress is shown in Figure 10. The crystal was subjected to a tensile stress which was raised slowly from zero to 2.6 MPa. Twinning occurred initially along the thin ribbon (T_1_) at a stress value of 2.3 MPa. This is the primary twin and was observed to grow with increasing stress. At 2.6 MPa a secondary twin system was initiated, intersecting the original twin at two positions (S_1_ and S_2_). At this point the stress was relaxed to zero and the optical micrograph shown in Figure 10a taken. In the time elapsed while taking the micrograph, both twins decreased slightly in size. The stress was then increased from zero and the twins began to grow larger at a stress of 1.0 MPa. At position S_3_ a third secondary twin is nucleated and a portion of the primary twin is seen to separate and propagate away from the remainder of the twin. Figure 10b was taken at a stress value of 1.3 MPa and shows this. At a stress of 2.3 MPa another primary twin (T_2_) is nucleated and grows under constant stress (Figure 10(c)). Figure 10d shows the crystal surface 30 minutes after the stress has been released to zero. Over this period the twins became smaller and eventually disappeared, leaving the crystal surface in its original condition. The stress was again raised from zero. Both twins nucleated at the same position as before, although at the much lower stress value of 0.7 MPa (Figure 10(e)). In Figures 10 f) and (g) the existing twins grow in size and more twins are nucleated as the stress is increased further. At a stress of 1.2 MPa another, secondary twin appears at S_4_ (Figure 10(h)). At this stress the crystal fractures. Figures 10(i) and (j) show the twins diminish in size as the stress is relaxed by failure. However, the larger twins do not disappear entirely, but shrink to a certain size and remain permanently.

 By observation of the twins on two {011} faces, the twinning planes were found to be (101) for the twins labelled T and $\left[\overline{1}01\right]$ for those labelled S. In both cases, the twins were observed due to the introduction of a surface step as the lattice is displaced by the twinning shear. In the case of the (101) twins, the elastic strain due to lattice distortion can also be discerned for some way into the bulk of the crystal. The twinning elements for the larger (101) twins are: K_1_(101); K_2_(001); n_1_$\left[\overline{1}01\right]$; n_2_[100]. The twinning shear strain was calculated to be 0.353 for the (101) twin plane.

## Discussion

Examination of the regions surrounding unetched and etched Vickers hardness impressions shows that deformation occurs by an extremely complex mechanism involving dislocation slip, twinning and fracture. In order to explain from theory the hardness phenomena associated with Vickers indentation in this material, all of these factors require to be considered. Although a relatively simple model based on the ERSS for Vickers indentation is available [37] it is unlikely to be useful here because of the extensive twinning and fracture. Nevertheless, the observed variation of VHN with crystal face and orientation can be understood in terms of the anisotropy of the crystal structure. The deformation systems which accommodate the indentation pressure lie in discrete crystallographic orientations. This is illustrated by the fact that the deformation patterns, although complex, reflect the symmetry of the lattice planes on which they occur. On (101) planes, the deformation patterns show two fold rotational symmetry, while on {011} and {110} planes they are asymmetric as expected for a structure with the space group P2_1_/n. By changing the orientation of the indenter with respect to the deformation system, either by indenting on different faces or at various surface orientations, the response of the solid will be altered resulting in variations in hardness. This is clearly illustrated by the difference in the nature of the deformation patterns obtained at various indentations.

The strain fields produced around pyramidal indentations are geometrically similar irrespective of their size. Thus at heavier loads, the dimensions of the hardness impressions and their associated deformation features are proportionately larger, but otherwise identical. Consequently, the observed independence of Vickers hardness with load is as expected.

The decrease in hardness on the (010) face observed with increasing temperature can be attributed to a gradual reduction in the intermolecular bonding due to the thermal expansion of the lattice.

Dislocation slip is considered to be more important, and possibly the dominant mode of deformation during Knoop indentation. This is evident from several observations. First, there is a considerable reduction in the incidence of cracking compared with that associated with Vickers indentations. Second, dislocation slip as measured by the etch pit arrays surrounding the indentation impressions, is shown to be much more extensive when the Knoop indenter is used, in comparison with the Vickers. Both these factors result from the blunter geometry of the Knoop indenter which serves to inhibit brittle fracture and promote plastic deformation. Third, in the majority of Knoop indentations the degree of twinning is also reduced, except perhaps in the case of indentations on the {110} face.

According to the models of Daniels and Dunn, the observed anisotropy in Knoop hardness is determined mainly by the orientation of the indenter with respect to the operative slip systems, taking into account that the comparison between Knoop hardness and ERSS value must be limited to those orientations at which the same slip system is active on a given face. A reasonable correlation exists for the (010) face, particularly considering that relatively minor variations in the ERSS are involved. Support is also given to the model and the slip systems believed to participate in the deformation process from the microscopic observation of etch pit patterns. Although the data is more limited for the {011} face, again it appears that directions which correspond to the lowest values of the ERSS are hardest and those with the highest ERSS are softest. Twinning has been considered as a potentially important mechanism, but microscopic examination reveals that the process is not particularly extensive, and that on the (010) face there is no correlation between the appearance of twin lamellae and the ERSS, except perhaps for alignment along the $\left[10\overline{1}\right]$ direction. This assumes of course that the twinning system is (101) $\left[10\overline{1}\right]$. The experimental results and observations therefore support the view that slip is the major mechanism leading to hardness anisotropy and determining the deformation mechanism during indentation of the (010) and {011} faces. On the {110} face, the observed slip system found to be active does not agree with the predictions of the Daniels and Dunn model. There is also little orientational dependence of hardness in spite of relatively large variations in ERSS (compared to the (010) face for example). On this face twinning is more extensive than is observed on the (010) and {011} faces, and probably provides a significant contribution to the deformation process.

Palmer and Field [22] have stated that mechanical twinning occurs in crystals when subjected to shear stress parallel to the (101) plane, i.e. the compositional plane of the twin. From their description of the twinned morphology, the twinning elements are determined as K_1_; (101), n_1_; $\left[10\overline{1}\right]$, K_2_; (100), n_2_; [001] with a twinning shear of 0.353. Since K_1_, K_2_, n_1_ and n_2_ are all rational, such a twin is a compound twin. Observations on the (010) and {110} faces would appear to confirm this view regarding the nature of the mechanical twin. The observations made in reflected light around indentations on the {110} face reveal steps at the surface due to twin lamellae, whereas no such steps are observed on the (010) face where twins can be viewed under transmitted light. These observations are totally in keeping with a twinning direction of $\left[10\overline{1}\right]$. However, the situation is not quite so straightforward on a {011} face where consideration of the crystal structure suggests that twins would emerge at this surface and produce steps as on the {110} face. There is no significant evidence for steps at any of the orientations used in these studies, although the twins are clearly present when the indented sample is viewed in transmitted light. It is expected that steps would be absent only if the direction of twinning is $\left\langle \overline{1}11\right\rangle$. These observations together with those already discussed with regard to ERSS and hardness measurements suggest that twinning may be more complicated than previously anticipated and that a more thorough investigation is called for.

The Peierls-Nabarro model [38] predicts that dislocation glide will take place in the direction of the shortest lattice translation on those planes having the largest interplanar spacing. The five smallest Burgers vectors and the interplanar spacing of the lowest index planes, taking into account the halving due to the diagonal glide plane are listed in Table 5. These show that the (001) [100] and (101) $\left[10\overline{1}\right]$ slip systems determined from the indentation and etching studies are different to those expected from the model.

**Table 5:** **A comparison of hardness values for other organic systems**

**Material**	**Face**	**Range of H**_**V**_ **(MPa)**	**Range of H**_**K**_ **(MPa)**
TNT (Unpublished observations)	{001}	219.7-220.6	201.0-235.4
Paracetamol [4]	{001}	352.1-392.3	235.4-411.9
	$\left\{00\overline{1}\right\}$	382.4-442.3	304.0-456.0
	{110}	352.1-392.3	353.0-402.1
	{011}	361.8-402.1	
RDX [7]	{210}	371.7-382.4	313.8-430.5
PETN [7]	{110}	147.1-166.7	127.5-245.2
	{101}	156.9-166.7	137.3-175.5
Adamantane [40]	{222}	40.2-50.0	40.2-59.8
HMX	{010}	323.6-362.8	313.8-481.5
	{011}	313.8-343.2	343.2-372.7
	{110}	304.0-313.8	372.7-451.1

Since cleavage involves a splitting of the crystal along specific planes, it will preferentially take place parallel to those planes which are weakly bonded. Calculation of the values for the interplanar spacing for the (011) and (020) planes (d_011_ = 0.6021 nm and d_020_ = 0.5525 nm) shows that they are amongst the three most widely separated planes and are therefore likely to be held by weak interactions and parted easily. The cleavage cracks are probably simple tensile cracks. There is no evidence to suggest that they are formed as a consequence of dislocation interactions.

Palmer et al. [24] have observed twinning which takes place on the (101) plane during deformation by compression. At a certain critical stress, elastic twins were formed, which increased in size and number with increasing stress. Upon removal of the load, the twins were observed to disappear. Further increase in stress resulted in many of the twins becoming permanent and led eventually to crystal, fracture along undefined directions. The origin of the elastic twinning was attributed to the ‘locked-in’ stresses which occur with shearing of the lattice.

The twinning behaviour under tension described here is similar to that noted by Palmer et al. in compression. In this study, two types of twin were observed. The twin which formed initially (primary twin) is identical to that observed previously. The twin nucleated at slightly higher stress (secondary twin) is strained elastically along the same direction in which fracture was found to occur. As before, increasing the stress resulted in the growth of both twins and the nucleation of other new twins. When a twin was nucleated at a certain stress value, which was held constant, the twin continued to grow. This means that the stress required for nucleation is greater than that needed for growth. Repeated nucleation and relaxation of the twin resulted in the re-introduction of the twin at successively lower stress values and eventually resulted in one of the twins becoming permanent and finally crystal fracture.

The highly brittle nature of β-HMX is illustrated by the fact that the primary twin is nucleated instantaneously in a thin lamellae which runs throughout the crystal, with boundaries at the crystal surface. A more plastic material would produce twins embedded in a crystal matrix, with the lattice distortion accommodated by slip. The secondary twin consists of a thin lamellae contained in the crystal bulk, intersecting the surface on two {011} faces. In this case, the lattice distortion is taken up by elastic strain. At higher loads, in the absence of slip, the lattice will be unable to accommodate the greater distortion and fracture will result. Although observed by Palmer et al. [24], this was not seen here. This is possibly due to the differences in ERSS for twinning in tension and compression.

Repeated twin nucleation always occurred in the same position, suggesting that some lattice defect acts as a site for twin nucleation. Whether due to an impurity acting as a stress concentrator or a dislocation, as in a pole dislocation twinning mechanism, is unknown. Observations of the present type, looking solely at the twin morphology provides only limited information. More detailed investigations of the processes taking place in the bulk of the crystal are ideally suited to X-ray topography (XRT) as has been demonstrated for PETN. Tensile testing in conjunction with XRT will yield information on the defects active in twinning [7]. Synchrotron radiation topography also makes possible dynamic studies, observing the motion and growth of the twins as it occurs. This experiment will be repeated ‘in situ’ during XRT in order to investigate more fully the nature of the twinning in the bulk of the crystal.

Use of the tensile stage with XRT will be extended to other materials to study the role of defects and, in particular, dislocations during plastic deformation in a similar manner.

## Conclusions

Microhardness indentation techniques have been used to investigate the mechanical deformation behaviour at the three predominant habit faces of β-HMX. Both Vickers and Knoop indenters have been used to determine the variations in hardness with orientation, load and temperature. Indentations were etched to identify the active slip systems and the measured hardness was compared with the hardness predicted from ERSS calculations. The Vickers hardness number (VHN) was found to vary in the range of 304–363 MPa depending on crystal face and surface orientation, however the VHN was found to be independent of load (in the range 15 g-35 g), thus implying that the deformation mechanism remains constant under these conditions. With varying temperature, the VHN decreased as would be expected due to thermal expansion, of the lattice up to a temperature of 378 K, indicating that there was no change in deformation mechanism up to this point. A greater anisotropy was found for the KHN, varying between 314–482 MPa depending on crystal face and orientation.

The two slip planes revealed from indentation etching studies are (001) and (101) and the most likely Burgers vectors for dislocation glide on these planes as deduced from simple structural considerations are [100] and $\left[10\overline{1}\right]$ respectively. Comparison of the Knoop hardness anisotropy with that predicted by ERSS calculations using the (001) [100] and (101) $\left[10\overline{1}\right]$ slip systems showed reasonable agreement, but it proved difficult to judge conclusively at this stage whether or not dislocation slip controls the deformation process during Knoop indentation. Twinning was observed to occur during deformation. The extent of twinning depended on the crystal face that was indented and on the orientation of the indenter. Although observations on the (010) and {110} faces supported a (101) $\left[10\overline{1}\right]$ twin system, little correlation was found between the calculated ERSS values and the observed extent of twinning during indentation of the (010) face. Indentation of the {011} face proved anomalous in that a twinning direction of $\left\langle \overline{1}11\right\rangle$ was suggested.

In comparison to other secondary explosives, HMX is considered to be brittle and the hardness numbers can be compared to other organic solids (Table 5) and the values are comparable to those of other brittle systems, such as paracetamol [4]. It has been shown previously [22] that single crystals of HMX deform by twinning prior to fracture when loaded in compression. However, the work described here clearly shows that under conditions developed beneath a Vickers or in particular a Knoop indenter, dislocation slip plays an important role in accommodating the local deformation stresses. Since microhardness indentation testing simulates the conditions which might be experienced by an explosive crystallite during impact more closely than a conventional deformation experiment [39], the results obtained here demonstrate the importance of this technique in the study of the impact detonation of explosives.

## Additional file

Additional file 1:
**Projections necessary for constructing an ERSS curve for Knoop indentations on a (010) face of HMX.**
