# Supplementary material for: Mechanical properties of β-HMX
Source: Chem Cent J. 2015 Apr 18;9:22. doi: 10.1186/s13065-015-0091-6 (PMC4439438; doi:10.1186/s13065-015-0091-6)
Supplement: Additional file 1: — Projections necessary for constructing an ERSS curve for Knoop indentations on a (010) face of HMX. [file 13065_2015_91_MOESM1_ESM.docx]

**Effective Resolved Shear Stress Calculations**

According to Daniels and Dunn [31], the ERSS associated with a particular slip system is related to the tensile component F of the applied force acting along each face of the indenter and is given by;

ERSS = $\frac{F}{A}$ cos λ cos φ cos ψ

Where A is the area of the specimen undergoing deformation, λ is the angle between the tensile stress axis and the slip direction and φ is the angle between the tensile stress axis and the slip plane normal. The cos ψ term, where ψ is the angle between horizontal axis H parallel to the indenter face and the axis of rotation of the slip system during deformation, arises from material constraints associated with dimensional changes accompanying deformation. It is only possible to determine relative values of ERSS at different orientations of the indenter, since the tensile component of the force remains unknown. These values depend therefore, solely on the geometrical relationship contained in equation 1 (i.e. cos λ cos φ cos ψ).

The Brookes, O’Neill and Redfern equation [32] is similar to that given by Daniels and Dunn, but includes an additional constraint term, sin γ, where γ is the angle between the axis H and the slip direction.

ERSS = $\frac{F}{A}$ cos λ cos φ (cos ψ + sin γ)

A full explanation of how the analysis is carried out using a stereographic projection is to be found in the original paper of Daniels and Dunn. The example illustrated in the following diagram shows the projections necessary for constructing an ERSS curve for Knoop indentation on a (010) test face of HMX where the active slip systems are assumed to be (101)$\text{[10}\bar{\text{1}}\text{]}$ and (001)[100] and the indenter is aligned along the designated zero direction. In practice the stereographic projection forms a template which can be superimposed at any orientation onto the stereographic projection for the test surface. Loci for directions parallel to each face of the indenter are represented by the arcs of circles AB, BC, CD and DA. The lines of greatest slope along which the tensile shearing force is presumed to act are found at T_1_, T_2_, T_3_ and T_4_. The cos λ cos φ cos ψ or cos λ cos φ (cos ψ + sin γ) terms were calculated for each of the four faces and the ERSS value expressed as an average of these four terms.

$\text{101101}$
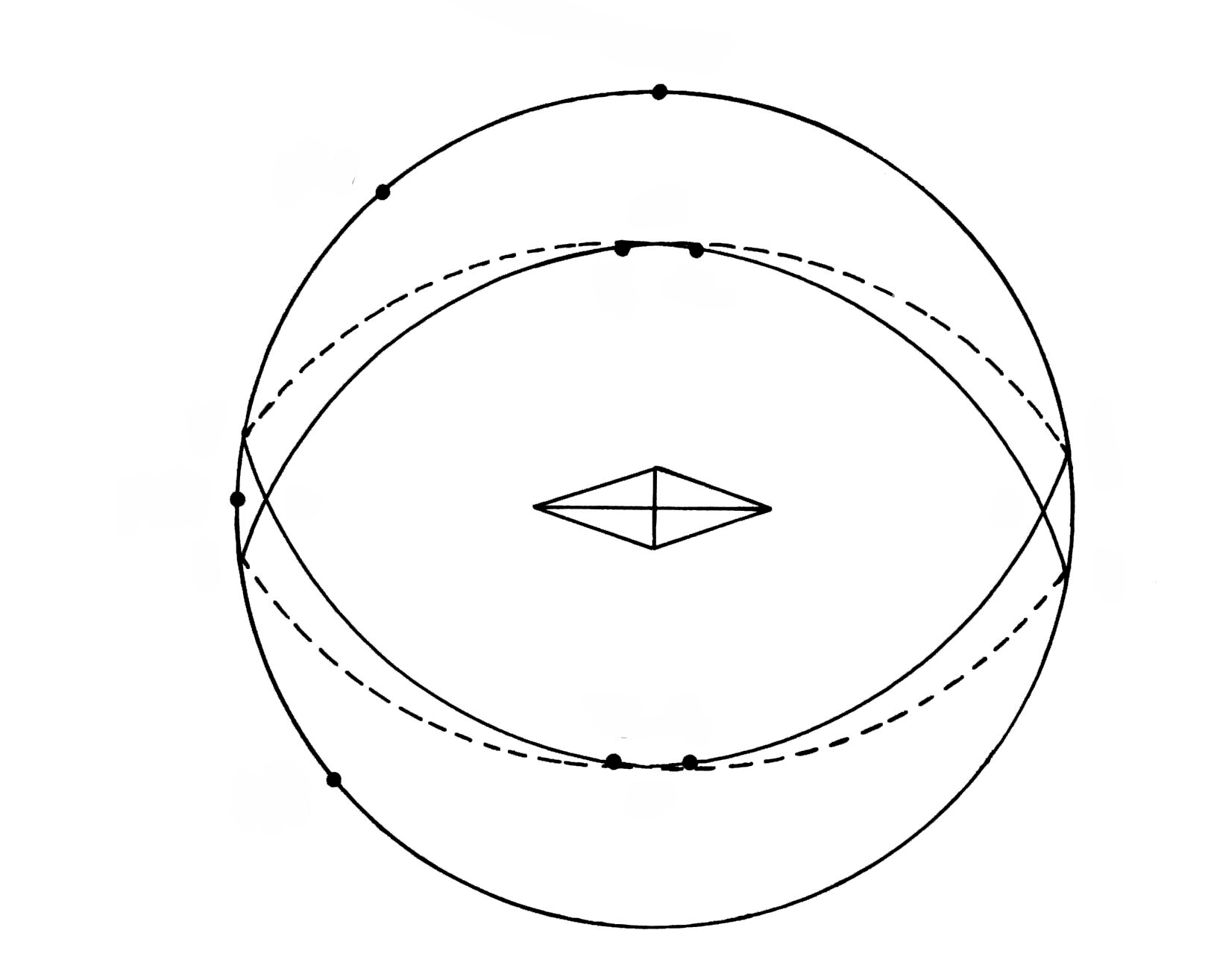


**[100]**

**(101)**

**(001)**

**H**

**H**

**H**

**H**

**T_4_**

**T_3_**

**T_2_**

**T_1_**

**D**

**B**

**C**

**A**
